# Understanding as Designed Projection

**DOI:** 10.1007/s42113-026-00299-3

**Published:** 2026-04-15

**Authors:** Yinqi Huang

**Affiliations:** School of Psychology, Georgia Institute of Technology, Atlanta, GA United States

## Abstract

Computational models can predict and generalize while leaving mechanistic questions unanswered. In their recent commentary, Shiffrin et al. argue that this gap gives rise to “illusions of understanding” in sciences, particularly when successful inference is treated as explanation. In this commentary, I focus on one source of such illusions emphasized by the authors: the difficulty of deduction. The authors propose that such illusions arise because deductions, even when correctly formalized, are often difficult to understand in probabilistic, chaotic, or high-dimensional system. Here I reconsider this conclusion by arguing that this limitation is better understood as a consequence of a designed projection under partial observability. Scientific measurement relies on compressing complex phenomena through experimental design, creating an information bottleneck. From an information-theoretic perspective, no analysis can recover structure that was never measured. Drawing on recent work on epiplexity, I further argue that understanding emerges from the extraction of learnable structure from data, shaped by both data collection and analysis choices. In this view, scientific progress is driven by improving and switching projection spaces through new measurements, task designs, and models that make previously inaccessible structure learnable.

Shiffrin et al. (2026) offer a timely reminder that scientific practice is vulnerable to overconfidence. A model can fit, predict, and even generalize while leaving core mechanistic questions unresolved. This caution is particularly relevant in an era when high-capacity models such as large language models can achieve impressive predictive performance with limited interpretability. The authors argue that prediction is not identical to understanding the mechanisms, against the rhetorical drift in empirical science that treats successful inference within a chosen analytic framework as if it constituted a complete explanation of the underlying phenomenon. They further argue that such illusions of understanding arise in part from the difficulty of deduction: even when correctly formalized, deductions in probabilistic, chaotic, or high-dimensional systems can be difficult to verify or interpret, and thus may give a false sense of understanding.

In this commentary, I reconsider this argument by situating the difficulty of deduction within the broader structure of scientific inference. Scientific inquiry proceeds by first compressing a phenomenon into a falsifiable hypothesis through selected measurements, and then inferring underlying causes from the measured data. However, a single study cannot represent every relevant variable, timescale, or causal pathway. As a result, even when inference is internally consistent, compressing a complex system inevitably leads to information loss, and deductions drawn from the compressed representation can be mistaken for deductions about the full system. This is a genuine concern and limitation. However, describing it as an “illusion” risks implying that scientific understanding is illusory. Here I propose a reinterpretation. The core limitation is not illusion, but projection in the sense of partial observability under compression. This echoes perspectives in the philosophy of science that scientific observation and inference are not theory-neutral but are shaped by underlying conceptual frameworks and methodological lenses (Kuhn, 1994; Lakatos, 1970), with Lakatos (1970) further characterizing scientific understanding as a form of “rational reconstruction.” From this perspective, scientific understanding is not an illusion but a designed projection. Thus, scientific progress is driven by improving and switching projection spaces.

A helpful metaphor for this reinterpretation is Plato’s Allegory of the Cave. In the allegory, prisoners observe shadows cast on a wall and come to treat those shadows as the world itself. Importantly though, those shadows are not hallucinations. They are lawful projections of real objects under constraints imposed by geometry, lighting, and the observers’ position. The limitation is not that the observations are false, but that they are partial and viewpoint-dependent. This metaphor maps naturally onto scientific measurement and deduction. Data are not reality itself. Rather, data are reality as observed through a measurement. What we record is inevitably affected by experimental design, which constrains what variations can be expressed, instrumentation, which constrains what signals can be detected, and variable definitions, which constrain what counts as an observable. These choices are the means by which complex phenomena become compressible into a form that allows for scientific testing. Thus, the core issue may be better described as projection and selection rather than hallucination. Researchers select a cross-section of a phenomenon and risk mistaking that cross-section for the whole object.

This limitation can be expressed more explicitly in information-theoretic terms. Scientific inquiry necessarily transforms a complex phenomenon into a data stream that is finite and compressed, because experimenters must select what to record, when to record it, and with what resolution. The resulting observations therefore act as an information bottleneck. Only a subset of the structure present in the underlying phenomenon can make it to the dataset, and the rest becomes inaccessible under the current measurement regime. From this perspective, the difficulty of deduction is not a cognitive failure, but a structural constraint imposed by the information transmitted through measurement and by the computational limits of the learner. The data processing inequality makes this constraint precise in a probabilistic sense. If the phenomenon * X* generates observations * Y*, and an estimate $\:\widehat{X}$ based on * Y*. Thus, the mutual information between * Y* and $\:\widehat{X}$ cannot exceed the mutual information between * X* and * Y*, that is, inference cannot increase mutual information about * X* beyond what the observations * Y* contain. In other words, no post hoc transformation of the observed data alone can increase information about the phenomenon beyond what is contained in the observations, unless additional assumptions, priors, or new observations are introduced.

Recent work by Finzi et al. (2026) on epiplexity strengthens this argument by distinguishing two types of information in data. Traditional measures such as Shannon entropy quantify uncertainty, but they do not distinguish random unpredictability from learnable structure, and they implicitly treat the observer as computationally unbounded. In contrast, epiplexity characterizes how much structure a computationally bounded learner can extract and compress into an internal model. This distinction matters because high-entropy data can be informationally rich while remaining structurally useless for learning, whereas data with moderate entropy can support strong generalization if it contains compressible content. In this view, understanding emerges when a learner succeeds in converting part of the dataset into a compact internal description, but the strength of the resulting deductions is conditional on what made the data computationally accessible in the first place. The illusion, therefore, is not that scientists invent patterns from nothing, but that they may mistake the learnable structure captured by partial dataset and models for the full structure of the phenomenon.

Thus, the cave metaphor can be sharpened further by noting that the measurement bottleneck is not a passive constraint, but actively designed by scientists. Researchers choose what counts as a variable, what counts as a trial, what counts as noise, and what counts as signal, and these choices determine what information enters the dataset and what remains inaccessible. From information-theoretic perspective, experimental design, therefore, is a primary factor of which structures become learnable under bounded computation. Thus, scientific progress can then be reframed as progress in selecting appropriate representations, achieved by developing new measurement tools, new task designs, and new analysis that make previously inaccessible structures extractable. In this light, debates about “illusions of understanding” are best addressed by asking which measurement and representational choices make certain deductions learnable, which make others unlearnable, and what would expand the learnable structure.

## Data Availability

Not applicable. This article is a commentary and does not report original empirical data.

## Code Availability

Not applicable. No code was generated for this commentary.

## References

[CR1] Finzi, M., Qiu, S., Jiang, Y., Izmailov, P., Kolter, Z. J, & Wilson, G. A (2026). From entropy to epiplexity: Rethinking information for computationally bounded intelligence. *arXiv*. 10.48550/arXiv.2601.03220

[CR2] Kuhn, T. S. (1994). *The structure of scientific revolutions* (2. ed., enlarged, 21. print). Univ. of Chicago Press.

[CR3] Lakatos, I. (1970). History of science and its rational reconstructions. *PSA: Proceedings of the Biennial Meeting of the Philosophy of Science Association*, *1970*, 91–136. 10.1086/psaprocbienmeetp.1970.495757

[CR4] Shiffrin, R. M., Stigler, S., & Keil, F. (2026). Illusions of understanding in the sciences. *PsyArXiv*. 10.31234/osf.io/37ps8_v1

